# 肺亚实性结节生长风险评估与精准管理研究进展

**DOI:** 10.3779/j.issn.1009-3419.2026.106.20

**Published:** 2026-06-20

**Authors:** Shulei CUI, Linlin QI, Jianwei WANG

**Affiliations:** 100021 北京，国家癌症中心/国家肿瘤临床医学研究中心/中国医学科学院北京协和医学院肿瘤医院影像诊断科; Department of Diagnostic Radiology, National Cancer Center/National Clinical Research Center for Cancer/Cancer Hospital, Chinese Academy of Medical Sciences and Peking Union Medical College, Beijing 100021, China

**Keywords:** 亚实性结节, 生长, 随访, 影像组学, 深度学习, 单细胞测序, Subsolid nodules, Growth, Follow-up, Radiomics, Deep learning, Single-cell sequencing

## Abstract

肺亚实性结节（subsolid nodules, SSNs）生长具有异质性。多数SSNs在长期随访观察过程中保持稳定或仅表现为缓慢生长，而少数病灶则在较短时间内迅速生长，甚至进展为浸润性腺癌（invasive adenocarcinoma, IAC）。SSNs的生长是其生物学行为演变的重要影像学表现，也是影响随访策略制定和干预时机选择的关键依据，如何精准评估其生长风险已成为当前研究关注的重点。本文从SSNs的自然史及临床随访管理的难点问题出发，系统梳理了SSNs生长风险评估在影像学、影像组学和深度学习领域的研究进展，并进一步总结相关分子生物学证据，旨在为SSNs生长风险的精准评估及个体化管理策略的制定提供理论依据。

## Abstract (en)

Pulmonary subsolid nodules (SSNs) exhibit heterogeneous growth patterns. Most SSNs remain stable or grow only slowly during long-term follow-up, whereas a small subset grows rapidly over a relatively short period. Some of these lesions may ultimately progress to invasive adenocarcinoma (IAC). Growth is an important imaging manifestation of evolving biological behavior in SSNs. It also serves as a key determinant of surveillance strategies and the timing of intervention. Accordingly, accurate assessment of growth risk has become a major focus of current research. Starting from the natural history of SSNs and the challenges associated with their clinical surveillance and management, this review systematically examines advances in SSNs growth risk assessment across imaging, radiomics, and deep learning. It also summarizes relevant molecular biological evidence to inform accurate growth risk assessment and the development of individualized management strategies for SSNs.

据国际癌症研究机构GLOBOCAN数据库统计，肺癌的发病率及相关死亡率在全球恶性肿瘤中仍居前列^[1]^。近年来，随着低剂量计算机断层扫描（low-dose computed tomography, LDCT）筛查的广泛应用，肺亚实性结节（subsolid nodules, SSNs）的检出率明显增加。我国部分LDCT筛查数据^[2,3]^显示，SSNs在检出肺癌中的占比已逾80%。在影像学层面SSNs表现为肺窗内局灶性淡薄密度增高影，且未掩盖血管与支气管轮廓。根据实性成分的存在与否，SSNs可分为纯磨玻璃结节（pure ground-glass nodules, pGGNs）和部分实性结节（part-solid nodules, PSNs）；后者在影像学上表现为同时含有磨玻璃成分和实性成分，亦称混合磨玻璃结节（mixed ground-glass nodules, mGGNs）^[4,5]^。持续存在的SSNs通常被认为是肺腺癌早期病变的重要影像学表现，其病理谱系可涵盖非典型腺瘤样增生（atypical adenomatous hyperplasia, AAH）、原位腺癌（adenocarcinoma *in situ*, AIS）、微浸润腺癌（minimally invasive adenocarcinoma, MIA）以及浸润性腺癌（invasive adenocarcinoma, IAC）^[6]^。由于SSNs在自然病程、生长速率及恶性转化风险上表现出显著的生物学异质性，多数SSNs在长期随访观察过程中保持稳定或仅表现为缓慢生长，而少数病灶则在较短时间内迅速生长，甚至进展为IAC^[6-8]^。因此，明确SSNs的生长规律并制定合理的随访与干预策略，对提高肺癌早期精准诊疗水平、避免过度干预及改善患者预后具有重要意义。

临床实践中SSNs的临床管理仍高度依赖影像学随访。现有指南^[5,9-12]^主要根据结节大小和实性成分制定相应的随访与干预策略，在一定程度上规范了SSNs的临床管理。然而，对于“哪些SSNs更可能生长、生长速度如何、何时干预及随访持续时间”等关键临床问题，尚未形成统一认识。由于SSNs自然病程较长，且不同病灶生物学行为差异明显，现行缺乏个体化的随访策略极易使临床陷入双重困局：一方面一刀切式的长期随访引发患者的长期心理焦虑并导致过度干预；另一方面，频繁的CT复查不仅加剧了医疗资源的消耗，还带来了潜在的累积辐射风险^[13]^。研究^[14]^数据表明，LDCT筛查中约18.5%的肺癌病例可能属于过度诊断，其中非小细胞肺癌（non-small cell lung cancer, NSCLC）的过度诊断率高达22.5%。对于长期稳定或生长缓慢的SSNs，适度放宽随访间期、减少不必要的CT检查，有助于降低辐射暴露并优化医疗资源配置。因此，准确识别具有生长潜能的高危SSNs，并建立精准的风险分层及差异化随访管理策略，是当前肺SSNs临床管理中亟待解决的关键问题。

现阶段，针对SSNs生长风险评估的研究正在由传统影像逐步向定量化、多维度评估发展。传统影像学通过评估结节大小、形态特征以及实性成分的出现与增加来预测SSNs生长^[7,15]^，为SSNs的初步风险分层和随访管理提供了依据。但传统影像学评估在一定程度上依赖阅片经验，且对结节内部异质性改变及早期生物学进展的预测仍有限。近年来，影像组学和深度学习（deep learning, DL）技术逐渐应用于SSNs生长和进展预测。前者通过提取定量影像特征提升对病灶异质性的识别能力，后者则依托自动特征学习增强风险评估效能，推动了SSNs生长风险预测由传统经验判断向精准化、智能化方向发展^[16-18]^。随着单细胞测序等高通量技术的发展，研究者开始从分子改变及肿瘤微环境层面探讨SSNs发生、进展及生长差异的潜在机制，相关研究^[19,20]^提示，SSNs的演进过程伴随免疫微环境重塑及细胞间相互作用改变。这些研究为SSNs精准生长风险分层和个体化管理构筑了新基石。尽管如此，该领域的探索整体仍处于起步阶段，对于SSNs生物学演进的系统性认知依然匮乏，影像表型与分子特征之间的对应关系亦有待进一步阐明。

本文就SSNs的自然史及临床随访管理的难点问题，系统梳理了SSNs生长风险评估在影像学、影像组学和DL领域的研究进展，并进一步总结相关分子生物学证据，旨在为SSNs生长风险的精准评估及个体化管理策略的制定提供理论依据。

## 1 SSNs的自然生长规律与随访策略

### 1.1 SSNs生长的定义及常用评价指标

SSNs生长通常是指同一病灶在连续CT随访中出现超出测量误差且可重复确认的影像学进展，其表现包括结节最大径或平均径增大、体积增加、密度升高，以及新发或增大的实性成分；在能够进行可靠体积测量时，还可采用体积倍增时间（volume doubling time, VDT）量化其生长速率^[21,22]^。不同指南对SSNs生长的定义并不完全一致。Fleischner 2017^[5]^主要适用于偶发肺结节，强调随访中结节大小及实性成分变化的意义，通常认为结节中实性成分直径增加超过2 mm或者结节中出现新的实性成分为SSNs生长。Lung-RADS v2022^[9]^主要面向肺癌筛查场景，将结节平均径增加>1.5 mm（12个月内）作为生长标准之一，同时强调新发或增大的实性成分可提高风险分类。欧洲胸腔成像学会（European Society of Thoracic Imaging, ESTI）认为体积测量计算VDT是衡量生长速率的最佳方法。对于PSNs，其实性成分的体积分割往往不够准确。因此，推荐在这种情况下使用手动测量，并以>1.5 mm作为判断显著生长的阈值^[22]^。

### 1.2 SSNs的自然生长规律

SSNs通常呈现惰性的生物学行为。系统综述^[23]^显示，SSNs生长缓慢，其中pGGNs的平均VDT为669天，PSNs为536天。Ba等^[24]^纳入了138个pGGNs，其中25个在3年随访期间出现生长，113个保持稳定。Qi等^[6]^纳入95个SSNs进行长期随访发现，47个pGGNs和21个PSNs出现生长，生长SSNs的平均VDT为（1704.7±1493.7）天。Lee等^[25]^对在LDCT检查中前5年保持稳定的208个SSNs进行了长达10年以上的长期随访，发现13.0%的病灶出现生长，中位生长时间为103个月。Kim等^[26]^对135个pGGNs进行长期随访，中位随访时间为193个月，研究结果显示在10年内保持稳定的76个pGGNs中，3.9%在10年后出现生长。de Margerie-Mellon等^[27]^研究表明，表现为SSNs的肺腺癌整体生长及实性成分生长更符合指数增长模式，部分SSNs在随访过程中可出现明显进展。进一步分析显示，IAC的总体体积增长速率显著高于非侵袭性腺癌，且其VDT更短，这表明生长较快的SSNs往往具有更高的病理侵袭性和恶性潜能。

### 1.3 SSNs的随访策略

目前，国内外指南^[5,9-12]^针对SSNs的随访管理虽均以结节类型、大小及实性成分变化作为决策依据，但在生长定义及随访间隔等方面存在不同。因此，SSNs的随访策略尚未达成统一共识，且缺乏基于个体化风险分层的管理策略。pGGNs通常随访间隔较长，而PSNs因恶性风险更高，尤其当实性成分增大或新发实性成分时，普遍建议缩短随访间隔。Fleischner 2017^[5]^主要针对偶发性结节，对≤6 mm的SSNs无需常规随访，稳定SSNs建议随访至5年；AATS 2023^[11]^则认为SSNs虽多呈惰性过程，但仍具有长期进展风险，因此提出稳定5年后仍可继续低频长期随访，部分患者甚至应观察至10年以上；Lung-RADS v2022^[9]^和NCCN v1.2026^[10]^主要针对肺癌筛查人群，以pGGNs为例，Lung-RADS以30 mm作为随访界限，而NCCN则采用20 mm作为随访界限；中国肺癌筛查与早诊早治指南^[12]^同样立足筛查场景，整体策略偏积极，如基线pGGNs≥8 mm即建议3个月复查。国内外各指南随访策略对比见表1^[5,9-12]^。

**表 1:** 国内外主要指南对肺亚实性结节管理策略的比较

Nodule type/Criterion	Fleischner 2017^[5]^	Chinese guideline 2021^[12]^	Lung-RADS v2022^[9]^	AATS 2023^[11]^	NCCN v1.2026^[10]^
pGGNs	<6 mm: no routine follow-up; ≥6 mm: confirm at 6-12 months, then annually until 5 years	<8 mm: annually; 8-15 mm: 3 months ≥15 mm: short-term follow-up/MDT	<30 mm: 12 months; ≥30 mm: 6 months	≥6 mm: confirm at 6 months, then every 12-24 months for at least 5 years	<20 mm: annually; ≥20 mm: 6 months
PSNs	≥6 mm: confirm at 3-6 months	<6 mm: next year; 6-15 mm: 3 months; ≥15 mm: biopsy/PET/CT	≥6 mm with solid component <6 mm: 6 months; Solid component ≥6 mm and <8 mm: 3 months; Solid component ≥8 mm: diagnostic evaluation	Surveillance interval usually ≤12 months; Escalate management if the solid component increases or growth occurs	≥6 mm with solid component <6 mm: 6 months; Solid component ≥6 mm and <8 mm: 3 months; Solid component ≥8 mm: diagnostic evaluation
Growth definition	Increase in diameter ≥2 mm	Increase in diameter ≥2 mm	Mean diameter increase >1.5 mm within 12 months	Increase in diameter >1.5 mm; New solid component or increase in solid component >1.5 mm	Increase in diameter >1.5 mm; increase in solid component>1.5 mm

pGGNs: pure ground-glass nodules; PSNs: part-solid nodules; Lung-RADS: Lung Imaging Reporting and Data System; AATS: American Association for Thoracic Surgery; NCCN: National Comprehensive Cancer Network; PET/CT: positron emission tomography/computed tomography.

## 2 SSNs的生长风险评估

### 2.1 SSNs生长相关临床因素及影像学特征

肺SSNs的生长是其生物学行为演变的重要影像学表现，也是影响随访策略制定和干预时机选择的关键依据。在临床特征方面，既往研究^[28]^表明，男性、既往肺癌病史、年龄>65岁是SSNs生长的独立危险因素。但Wang等^[29]^对201例T1型肺腺癌的分析显示，惰性结节组（VDT>600天）和非惰性结节组（VDT<600天），在年龄、性别及吸烟状态方面均无显著差异。这种不一致提示，临床特征更适合作为背景风险修饰因素，而不宜单独用于决定干预。

在影像学特征方面，初始直径较大、实性成分及形态不规则等被认为是SSNs生长的危险因素^[6,7,28]^。Ba等^[24]^经过3年随访发现，初始直径≥8.5 mm和形态不规则是pGGNs短期生长的独立危险因素。随访中的动态变化应作为风险升级的核心依据。相较于缓慢的直径增大，SSNs实性成分出现或增加与恶性进展及IAC密切关联，应触发更高级别的风险评估^[30]^。He等^[31]^通过大小与密度评估pGGNs的生长，结果表明密度增高能更早反映病灶生长。近年来，异质性磨玻璃结节（heterogeneous ground-glass nodules, hGGNs）逐渐受到关注。hGGNs通常表现为GGNs内部出现局灶性或弥漫性密度增高区域，但该区域的密度尚未达到纵隔窗可识别或可测量的真正实性成分标准，可被视为pGGNs向PSNs演变过程中的中间影像表型。Sun等^[32]^和Zhang等^[33]^的研究表明，与pGGNs相比，hGGNs具有更快的生长速度和更高的病理浸润风险。因此，对于随访中整体或局部密度持续升高、但尚未形成明确实性成分的结节，不宜仍按普通pGGNs进行风险判断，而应结合密度变化幅度、异质性分布及纵向演变趋势开展更高级别的风险评估。尽管临床及传统的影像学特征能在一定程度上预测SSNs的生长，但传统影像特征仍依赖人工判读，观察者间差异较大，也难以充分刻画结节内部异质性，有必要引入定量影像指标或影像组学方法，以提高风险分层的客观性和可重复性。

### 2.2 基于影像组学的SSNs生长风险预测模型

影像组学通过提取结节的三维空间结构、纹理特征和一阶/高阶统计学参数，实现了结节特征的高通量量化。真实世界中部分初筛疑似高危的SSNs会在随访中自行吸收消散，盲目随访不仅增加辐射剂量，更会引发患者焦虑。在初筛结节的早期分流方面，Jin等^[34]^构建了融合影像组学评分与临床特征的模型，用于预测初筛SSNs的消退或持续存在，显示出良好的预测效能[曲线下面积（area under the curve, AUC）=0.959]。该模型的预测一致性显著优于经验丰富的影像科医师。Sun等^[35]^进一步探讨了持续存在的SSNs，构建了整合影像组学与年龄、病灶位置和大小等因素的列线图，用于预测结节生长或长期稳定，训练集和验证集AUC分别为0.843和0.824。该模型更贴近持续性SSNs随访强度的分层需求，但预测效能相对有限，且仍缺乏独立外部验证。精准预测SSNs生长速率是制定个性化随访策略与确定干预时间节点的关键。Ma等^[16]^进一步将研究终点由是否生长转向生长速度，依据VDT将273个SSNs分为快速生长组（VDT≤400天）和缓慢生长组（VDT>400天），融合传统影像学征象与影像组学特征的模型在训练集和验证集的表现优于单独影像学或影像组学模型。然而，该研究仅纳入生长结节，且快、慢生长样本分布不均，因此不能用于判断基线稳定结节未来是否生长。类似地，Tan等^[36]^以生长速度为预测目标，整合了临床和放射学特征，建立了预测早期肺腺癌生长速率的列线图，验证集AUC约为0.78。该研究纳入经病理证实的早期肺腺癌，并同时包含GGNs和实性结节，其研究人群与筛查或随访中尚未定性的SSNs存在明显差异，因而模型的适用范围相对受限。

### 2.3 基于DL的SSNs生长风险预测模型

DL是人工智能（artificial intelligence, AI）领域的重要技术分支，其通过卷积神经网络（convolutional neural network, CNN）、Transformer等多层神经网络结构，实现了从原始影像体素到高阶语义特征的端到端自动提取与表征。与传统影像组学依赖人工定义特征不同，DL能够直接从原始影像中自动学习多层次特征，因此在捕捉复杂影像模式和挖掘潜在生物学信息方面具有独特优势。现有SSNs生长预测研究多基于首次检查获得的单时点CT信息构建模型，用于预测结节在后续随访中是否发生生长。Tang等^[37]^比较了4种DL模型在预测pGGNs生长中的表现，结果显示，基于基线CT构建的DenseNet_DR模型在内部验证队列和独立机构外部验证队列中的AUC分别为0.79和0.70。亚组分析显示，该模型对1年内生长结节的预测性能较高（AUC=0.855），但对较晚发生生长的结节预测能力下降，提示基线影像可能更易识别近期生长信号，而远期生长预测仍具有较大不确定性。值得注意的是，模型在外部验证中的性能低于内部验证，可能与随访时间、扫描参数及人群构成差异有关，反映了基线模型在跨中心泛化方面面临的挑战。Wang等^[29]^构建了基于首次CT的临床-影像特征的DL模型，用于识别T1期肺腺癌中的惰性生长结节（VDT>600天）并估计VDT，惰性结节判别模型在训练集和测试集中的AUC分别为0.7707和0.7700，VDT回归模型的决定系数分别为0.8008和0.6268。该研究为首次检查时评估结节生长趋势提供了思路，但其采用单中心随机划分的内部测试，且模型输入为预先提取的结构化特征，并非直接基于原始CT影像的端到端学习，因此仍需独立外部队列进一步验证。然而，SSNs的生长具有明显的时空演变特征，因此仅基于单一时间点构建的预测模型仍存在一定局限。为解决这一问题，Liao等^[17]^开发了基于孪生编码器的DL模型（SiamModel），整合连续两次CT扫描（T_t−1_至T_t_）的结节影像以预测后续以质量增加为判定标准的SSNs生长。双时相模型在内部验证集和独立外部测试集中的AUC分别为0.858和0.862；当仅输入单次CT时，相应数值分别为0.855和0.821。双时相输入在外部测试集中的性能提升更为明显，提示纵向信息可能有助于增强模型适应不同人群差异。然而，该研究外部测试集中仅包含9个生长结节，且生长与非生长样本明显不均衡，因此其泛化能力仍需在更大规模人群中验证。相关研究见表2^[17,29,37]^。

**表 2:** 深度学习模型预测肺亚实性结节生长的研究

Study	Research task	Model/Input information	AUC	Main limitations
Tang et al.^[37]^	Predicting pGGNs growth based on baseline CT	DenseNet-DR; single-phase baseline CT nodule images	Internal validation: 0.79 External validation: 0.70	Performance declined on external validation; only single-phase images were used
Wang et al.^[29]^	Identifying indolent nodules (VDT>600 d) and estimating VDT	Neural network; clinical and radiomic features extracted from the initial CT scan	Training set: 0.7707 Test set: 0.7700	Single-center, small-sample study; internal testing only; not an end-to-end imaging model
Liao et al.^[17]^	Predicting subsequent growth of SSNs using serial CT	SiamModel; longitudinal imaging information from two adjacent CT examinations	Internal validation: 0.858 External validation: 0.862	The external set included only 9 growing nodules; severe class imbalance; the growth threshold requires further validation

AUC: area under the curve; SSNs: subsolid nodules; VDT: volume doubling time.

既往针对SSNs病理分型的研究多以二分类为主^[38-41]^，近年来已有研究^[42-45]^开始进一步探索三分类模型的构建。Shao等^[43]^建立并比较了多种3D DL模型，通过对结节三维数据进行特征学习，实现SSNs的病理三分类。Pan等^[44]^并未采用单一的三分类网络直接对肺腺癌侵袭性进行分层，而是构建了一种仲裁策略的DL框架，模拟了临床多位阅片者一致性判读及分歧仲裁过程，从而提升了三分类任务，尤其是MIA识别的性能。上述研究表明，DL在SSNs病理三分类方面已形成一定研究基础，但现有模型主要针对病理类别进行横断面判别，其研究终点并非结节随访过程中的实际生长行为。因此，病理三分类模型的结果不能直接等同于生长风险分层。目前，基于纵向随访结局将SSNs划分为稳定、缓慢生长和快速生长等不同风险等级的DL研究仍相对有限，相关风险界值和标签定义亦缺乏统一标准。未来应依托多中心、大样本及长期纵向随访队列，统一生长判定标准和风险分层界值，并开展独立外部及前瞻性验证；同时加强模型校准、可解释性和临床净获益评价，以推动DL模型由病理类别识别进一步拓展至SSNs动态生长风险分层和个体化管理。

## 3 SSNs生长的潜在分子机制

### 3.1 SSNs生长进展相关驱动基因

SSNs最常见的是表皮生长因子受体（epidermal growth factor receptor, *EGFR*）突变^[46,47]^，且在亚洲人群中尤为突出。已有研究^[48]^表明，*EGFR*突变型NSCLC的VDT显著长于野生型（中位数：676* vs* 139天），提示*EGFR*突变相关病灶总体生长较为缓慢。然而，该研究并未对SSNs和实性结节进行区分，其结果不能直接外推至SSNs。Kobayashi等^[47]^针对SSNs研究发现，与*EGFR*、Kirsten大鼠肉瘤病毒癌基因同源物（Kirsten rat sarcoma viral oncogene homolog, *KRAS*）、间变性淋巴瘤激酶（anaplastic lymphoma kinase, *ALK*）及人表皮生长因子受体2（human epidermal growth factor receptor 2, *HER2*）均为阴性的结节相比，携带*EGFR*突变的SSNs更易出现生长。另有研究^[49]^显示，*EGFR* L858R突变与SSNs总体积、实性成分体积及直径显著关联。除*EGFR*外，*KRAS*等驱动基因可能与较强的生长及侵袭潜能有关，而Erb-B2受体酪氨酸激酶2（erb-b2 receptor tyrosine kinase 2, *ERBB2*）、V-raf鼠肉瘤病毒癌基因同源物B1（v-raf murine sarcoma viral oncogene homolog B1, *BRAF*）和丝裂原活化蛋白激酶激酶1（mitogen-activated protein kinase kinase 1, *MAP2K1*）突变型病灶相对惰性^[46]^。随着SSNs由低级别病变向IAC演化，驱动基因与抑癌基因共突变的重要性逐渐增加。例如，*EGFR*合并*TP53*突变可进一步促进SSNs向更高侵袭性状态进展^[46]^。Zhou等^[50]^发现，随着SSNs从AIS向IAC的病理升级，*EGFR*和RNA结合基序蛋白10（RNA-binding motif protein 10, *RBM10*）的突变率显著上升。*EGFR*/*RBM10*共突变患者的血红素代谢和胆固醇稳态通路显著上调，这些患者的总体生存率可能低于*EGFR*突变患者。上述结果表明，共突变事件可能通过重塑转录组和代谢通路活性，加速SSNs的侵袭性演化并影响预后。

### 3.2 SSNs进展中的微环境重塑

近年来，单细胞测序与空间组学研究为建立SSNs影像表型与分子特征之间的对应关系提供了新的证据。Zhang等^[51]^对20个PSNs进行空间转录组分析，其中4个匹配单细胞RNA测序，发现恶性细胞可分为MC1和MC2两种演化状态：MC1主要对应影像学磨玻璃成分及病理学贴壁生长区域，MC2则富集于实性和浸润性区域。CD74表达与实性成分比例呈负相关（Rho=-0.69, *P*<0.001），并与贴壁生长特征呈正相关。细胞互作分析显示，MIF-CD74配体-受体对在MC1区域的信号显著强于MC2区域，提示MIF-CD74介导的巨噬细胞-肿瘤细胞互作减弱可能参与免疫逃逸及实性成分形成。Ren等^[52]^对GGNs和部分实性结节型肺腺癌进行单细胞测序，发现前者具有更强的CXCL9^+^肿瘤相关巨噬细胞（tumor-associated macrophages, TAMs）与CD8^+^组织驻留记忆T细胞免疫互作，而随着浸润和转移进展，尤其在PSNs中，TREM2^+^/SPP1^+^ TAMs及其与肿瘤细胞的互作增强，提示CXCL9^+^与TREM2^+^ TAMs之间的功能失衡可能反映SSNs由免疫活跃向免疫抑制状态转换。Xiong等^[53]^进一步通过单细胞和空间转录组分析发现，早期磨玻璃样肺腺癌已存在明显的转录异质性；通过比较MIA与IAC标本，肿瘤细胞逐渐丧失肺泡I型和II型细胞分化特征，并通过上皮可塑性形成增殖型、免疫活跃型及过渡型细胞状态，其中KLF4/JDP2调控的肺泡II型向I型细胞过渡状态具有一定的肿瘤抑制作用。上述结果提示，CD74信号降低、CXCL9^+^/TREM2^+^ TAMs失衡、上皮去分化及细胞状态异质性可能成为SSNs生长和实性成分增加的候选生物学标志。

就精准管理而言，结节直径或体积缓慢增加但分子层面仍呈肺泡分化保留和免疫活跃特征者，可能更适合继续密切随访；而出现密度持续升高或实性成分增加，并伴上皮去分化及免疫抑制特征者，可能代表更高的浸润风险，应考虑缩短随访间隔或提前评估干预指征。未来可将上述分子指标与结节体积、密度、实性成分比例及其纵向变化相结合，构建具有生物学可解释性的多模态风险模型。值得注意的是，现有证据主要来源于手术标本的横断面比较，尚不能证明相关分子特征能够预测同一SSNs的后续生长，也不足以直接改变现行临床决策；其预测价值仍需在具有连续CT检查、长期随访及组织学或分子学验证的前瞻性队列中确认。

## 4 SSNs的早期干预与内科治疗探索

由于SSNs在生长速度、浸润潜能及分子生物学特征等方面存在明显异质性，其管理策略应建立在准确风险评估的基础上。临床可综合结节类型、大小、实性成分比例、形态学特征及动态变化，并结合患者年龄、吸烟史和肿瘤家族史等因素，对SSNs的进展及恶性风险进行评估，进而实施风险分层和差异化管理。对于长期稳定、无高危影像学征象的低风险SSNs，应以规范化影像随访为主，避免不必要的侵入性检查和过度治疗；对于结节增大、实性成分出现或增加，但尚未达到明确手术指征的中风险SSNs，可在密切随访的基础上，结合影像组学、分子检测等手段进一步评估其生物学行为，并探索适宜的早期干预方式；对于持续生长、实性成分明显增加的高风险SSNs，则应及时进行多学科评估，优先考虑手术切除或其他局部治疗。对于暂不适宜接受局部治疗的特定患者，探索以内科治疗延缓结节进展，可能成为分层管理策略的有益补充。目前，针对SSNs早期进展机制的研究逐渐从单纯观察转向以内科干预为导向的探索。已有研究尝试通过调控特定分子异常或肿瘤微环境改变，延缓SSNs向IAC发展的过程。

Tian等^[54]^研究发现HER2靶向治疗在中国青年早发性肺腺癌患者中具有显著临床疗效，提示针对驱动基因异常的精准治疗可能成为部分高风险早期肺腺癌患者的重要干预方向。Peng等^[19]^在小鼠模型中证实，抗白细胞介素-1β（anti-interleukin-1 beta, anti-IL-1β）抗体治疗可显著减少促炎性巨噬细胞，抑制肺泡祖细胞的扩增/转化，从而降低早期肺癌的肿瘤负荷。该研究表明针对肿瘤微环境的干预可能具有预防和治疗潜在价值。除了靶向治疗和微环境干预外，药物再利用策略也为SSNs的内科管理提供了新的研究思路。Ortmeyer Welch等^[55]^在回顾性队列研究中发现，合并2型糖尿病的持续性肺结节患者在使用钠-葡萄糖共转运蛋白2抑制剂（sodium-glucose cotransporter-2 inhibitors, SGLT2i）后，结节生长进展风险显著下降，且最终接受外科手术干预的比例低于对照组（3% *vs *13%）。这一结果表明，应用便利的口服药物可能在SSNs早期干预中具有潜在价值，但其因果关系、适用人群、干预时机及长期获益仍需通过前瞻性临床研究加以证实。

## 5 小结与展望

SSNs自然史具有显著异质性，不同病灶在生长速度、进展风险及侵袭性演化上存在差异。现行以结节大小和实性成分为依据的经验性随访策略，虽在临床上具有参考价值，但难以精确识别高危结节、判断生长时间点及干预时机。近年来，影像组学和DL技术的应用提高了对SSNs生长潜能的预测能力，而单细胞测序及空间多组学研究则揭示了驱动基因、微环境等分子特征在SSNs演化中的作用机制，为建立具有生物学可解释性的风险预测模型提供了基础。

现有研究表明多维特征在SSNs精准风险分层中具有重要临床价值。未来研究需要突破单一数据维度的限制，将高阶影像特征与空间多组学结合，建立具有高度生物学可解释性的多模态融合预测模型。同时，应针对现有AI模型多依赖回顾性数据的情况，开展前瞻性、多中心临床队列研究，全面验证其在真实世界中的适用性和临床价值。此外，还需探索基于风险分层的主动干预策略，包括基于风险分层的主动监测临床路径及开展针对惰性SSNs的药物治疗（如SGLT2i）的临床试验，以评估其作为早期干预的可行性。在临床管理中基于SSNs的生长风险评估有助于将随访和干预策略由单纯依赖结节形态和手术切除转向结合影像、分子特征的精准风险分层管理，从而更有针对性地制定个体化随访间隔和早期干预方案；同时，在临床实践中仍需平衡过度干预与恶性进展防范之间的关系。

## References

[1] Bray F, Laversanne M, Sung H, et al. Global cancer statistics 2022: GLOBOCAN estimates of incidence and mortality worldwide for 36 cancers in 185 countries. CA Cancer J Clin, 2024, 74(3): 229-263. doi: 10.3322/caac.21834 38572751

[2] Fan L, Wang Y, Zhou Y, et al. Lung cancer screening with low-dose CT: Baseline screening results in Shanghai. Acad Radiol, 2019, 26(10): 1283-1291. doi: 10.1016/j.acra.2018.12.002 30554839

[3] Zhang Y, Jheon S, Li H, et al. Results of low-dose computed tomography as a regular health examination among Chinese hospital employees. J Thorac Cardiovasc Surg, 2020, 160(3): 824-831.e4. doi: 10.1016/j.jtcvs.2019.10.145 31987625

[4] Godoy MC, Naidich DP. Subsolid pulmonary nodules and the spectrum of peripheral adenocarcinomas of the lung: Recommended interim guidelines for assessment and management. Radiology, 2009, 253(3): 606-622. doi: 10.1148/radiol.2533090179 19952025

[5] MacMahon H, Naidich DP, Goo JM, et al. Guidelines for management of incidental pulmonary nodules detected on CT images: From the Fleischner Society 2017. Radiology, 2017, 284(1): 228-243. doi: 10.1148/radiol.2017161659 28240562

[6] Qi LL, Wang JW, Yang L, et al. Natural history of pathologically confirmed pulmonary subsolid nodules with deep learning-assisted nodule segmentation. Eur Radiol, 2021, 31(6): 3884-3897. doi: 10.1007/s00330-020-07450-z 33219848

[7] Qi LL, Wu BT, Tang W, et al. Long-term follow-up of persistent pulmonary pure ground-glass nodules with deep learning-assisted nodule segmentation. Eur Radiol, 2020, 30(2): 744-755. doi: 10.1007/s00330-019-06344-z 31485837

[8] Cui S, Qi L, Li F, et al. Correlation analysis between growth heterogeneity and genetic mutations in resected subsolid lung adenocarcinoma based on long-term computed tomography (CT) follow-up. Transl Lung Cancer Res, 2026, 15(2): 30. doi: 10.21037/tlcr-2025-aw-1249 41808701 PMC12969182

[9] Lung-RADS 2022 assessment categories[EB/OL]. https://www.acr.org/clinical-resources/clinical-tools-and-reference/reporting-and-data-systems/lung-rads.https://www.acr.org/clinical-resources/clinical-tools-and-reference/reporting-and-data-systems/lung-radsAccessed May 07, 2026

[10] National Comprehensive Cancer Network. NCCN clinical practice guidelines in oncology:Lung cancer screening (version 1. 2026)[EB/OL]. https://www.nccn.org/https://www.nccn.org/.Accessed May 07, 2026

[11] Chen H, Kim AW, Hsin M, et al. The 2023 American Association for Thoracic Surgery (AATS) expert consensus document: Management of subsolid lung nodules. J Thorac Cardiovasc Surg, 2024, 168(3): 631-647.e11. doi: 10.1016/j.jtcvs.2024.02.026 38878052

[12] He J, Li N, Chen WQ, et al. China guideline for the screening and early detection of lung cancer (2021, Beijing). Zhongguo Zhongliu, 2021, 30(2): 81-111. 10.3760/cma.j.cn112152-20210119-00060 33752304

[13] Song J, Hwang EJ, Yoon SH, et al. CT screening challenges amid rising threat of lung cancer in individuals who have never smoked. Radiology, 2026, 318(1): e251305. doi: 10.1148/radiol.251305 41591250

[14] Patz EF Jr, Pinsky P, Gatsonis C, et al. Overdiagnosis in low-dose computed tomography screening for lung cancer. JAMA Intern Med, 2014, 174(2): 269-274. doi: 10.1001/jamainternmed.2013.12738 24322569 PMC4040004

[15] Borghesi A, Michelini S, Golemi S, et al. What’s new on quantitative CT analysis as a tool to predict growth in persistent pulmonary subsolid nodules? A literature review. Diagnostics (Basel), 2020, 10(2): 55. doi: 10.3390/diagnostics10020055 31973010 PMC7168253

[16] Ma ZJ, Ma ZX, Sun YL, et al. Prediction of subsolid pulmonary nodule growth rate using radiomics. BMC Med Imaging, 2023, 23(1): 177. doi: 10.1186/s12880-023-01143-x 37936095 PMC10629176

[17] Liao RQ, Li AW, Yan HH, et al. Deep learning-based growth prediction for sub-solid pulmonary nodules on CT images. Front Oncol, 2022, 12: 1002953. doi: 10.3389/fonc.2022.1002953 36313666 PMC9597322

[18] Wu XY, Hua MQ, Xia YF, et al. Research progress of radiomics and artificial intelligence in the growth prediction of pulmonary ground-glass nodules. Zhongguo Yixue Jisuanji Chengxiang Zazhi, 2025, 31(1): 141-145.

[19] Peng F, Sinjab A, Dai Y, et al. Multimodal spatial-omics reveal co-evolution of alveolar progenitors and proinflammatory niches in progression of lung precursor lesions. Cancer Cell, 2026, 44(2): 321-339.e13. doi: 10.1016/j.ccell.2025.10.004 41202811 PMC12980502

[20] Shang J, Jiang H, Zhao Y, et al. Differences of molecular events driving pathological and radiological progression of lung adenocarcinoma. EBioMedicine, 2023, 94: 104728. doi: 10.1016/j.ebiom.2023.104728 37506543 PMC10406962

[21] Prokop M, Schaefer-Prokop C, Jacobs C, et al. Aggressiveness-guided nodule management for lung cancer screening in Europe-justification for follow-up intervals and definition of growth. Eur Radiol, 2026, 36(1): 122-134. doi: 10.1007/s00330-025-11647-5 40593169 PMC12712097

[22] Snoeckx A, Silva M, Prosch H, et al. Lung cancer screening with low-dose CT: Definition of positive, indeterminate, and negative screen results. A nodule management recommendation from the European Society of Thoracic Imaging. Eur Radiol, 2026, 36(1): 135-147. doi: 10.1007/s00330-025-11648-4 40593170 PMC12711968

[23] Jiang B, Han D, van der Aalst CM, et al. Lung cancer volume doubling time by computed tomography: A systematic review and *meta*-analysis. Eur J Cancer, 2024, 212: 114339. doi: 10.1016/j.ejca.2024.114339 39368222

[24] Ba W, Jiang C, Jiang Y, et al. Exploring the risk factors for the growth of pure ground-glass nodules in a 3-year follow-up period. Quant Imaging Med Surg, 2025, 15(5): 3923-3930. doi: 10.21037/qims-24-2086 40384712 PMC12082577

[25] Lee HW, Jin KN, Lee JK, et al. Long-term follow-up of ground-glass nodules after 5 years of stability. J Thorac Oncol, 2019, 14(8): 1370-1377. doi: 10.1016/j.jtho.2019.05.005 31085340

[26] Kim BG, Nam H, Hwang I, et al. The growth of screening-detected pure ground-glass nodules following 10 years of stability. Chest, 2025, 167(4): 1232-1242. doi: 10.1016/j.chest.2024.09.037 39389342

[27] de Margerie-Mellon C, Ngo LH, Gill RR, et al. The growth rate of subsolid lung adenocarcinoma nodules at chest CT. Radiology, 2020, 297(1): 189-198. doi: 10.1148/radiol.2020192322 32749206

[28] Liang X, Liu M, Li M, et al. Clinical and CT features of subsolid pulmonary nodules with interval growth: A systematic review and *meta*-analysis. Front Oncol, 2022, 12: 929174. doi: 10.3389/fonc.2022.929174 35860567 PMC9289285

[29] Wang B, Zhang H, Li W, et al. Neural network-based model for evaluating inert nodules and volume doubling time in T 1 lung adenocarcinoma: A nested case-control study. Front Oncol, 2023, 13: 1037052. doi: 10.3389/fonc.2023.1037052 37293594 PMC10244560

[30] Kim BG, Um SW. A narrative review of the clinical approach to subsolid pulmonary nodules. Ann Transl Med, 2023, 11(5): 217. doi: 10.21037/atm-22-5246 37007560 PMC10061480

[31] He Y, Xiong Z, Zhang J, et al. Growth assessment of pure ground-glass nodules on CT: Comparison of density and size measurement methods. J Cancer Res Clin Oncol, 2023, 149(12): 9937-9946. doi: 10.1007/s00432-023-04918-5 37249644 PMC11797657

[32] Sun JD, Sugarbaker E, Byrne SC, et al. Clinical outcomes of resected pure ground-glass, heterogeneous ground-glass, and part-solid pulmonary nodules. AJR Am J Roentgenol, 2024, 222(5): e2330504. doi: 10.2214/AJR.23.30504 38323785 PMC11161307

[33] Zhang Z, Zhou L, Min X, et al. Long-term follow-up of persistent pulmonary subsolid nodules: Natural course of pure, heterogeneous, and real part-solid ground-glass nodules. Thorac Cancer, 2023, 14(12): 1059-1070. doi: 10.1111/1759-7714.14845 36922372 PMC10125786

[34] Jin L, Liu Z, Sun Y, et al. Prediction of pulmonary ground-glass nodule progression state on initial screening CT using a radiomics-based model. Respirology, 2026, 31(1): 73-81. doi: 10.1111/resp.70115 40916158

[35] Sun Y, Ma Z, Zhao W, et al. Computed tomography radiomics in growth prediction of pulmonary ground-glass nodules. Eur J Radiol, 2023, 159: 110684. doi: 10.1016/j.ejrad.2022.110684 36621209

[36] Tan M, Ma W, Sun Y, et al. Prediction of the growth rate of early-stage lung adenocarcinoma by radiomics. Front Oncol, 2021, 11: 658138. doi: 10.3389/fonc.2021.658138 33937070 PMC8082461

[37] Tang Y, Li M, Lin B, et al. Deep learning-assisted development and validation of an algorithm for predicting the growth of persistent pure ground-glass nodules. Transl Lung Cancer Res, 2023, 12(12): 2494-2504. doi: 10.21037/tlcr-23-666 38205216 PMC10775010

[38] Xu Y, Li Y, Yin H, et al. Consecutive serial non-contrast CT scan-based deep learning model facilitates the prediction of tumor invasiveness of ground-glass nodules. Front Oncol, 2021, 11: 725599. doi: 10.3389/fonc.2021.725599 34568054 PMC8461974

[39] Park S, Park G, Lee SM, et al. Deep learning-based differentiation of invasive adenocarcinomas from preinvasive or minimally invasive lesions among pulmonary subsolid nodules. Eur Radiol, 2021, 31(8): 6239-6247. doi: 10.1007/s00330-020-07620-z 33555355

[40] Chen X, Feng B, Chen Y, et al. A CT-based deep learning model for subsolid pulmonary nodules to distinguish minimally invasive adenocarcinoma and invasive adenocarcinoma. Eur J Radiol, 2021, 145: 110041. doi: 10.1016/j.ejrad.2021.110041 34837794

[41] Qi K, Wang K, Wang X, et al. Lung-PNet: An automated deep learning model for the diagnosis of invasive adenocarcinoma in pure ground-glass nodules on chest CT. AJR Am J Roentgenol, 2024, 222(1): e2329674. doi: 10.2214/ajr.23.29674 37493322

[42] Wan Q, Zou Q, Sun C, et al. CT-based radiologic ternary classification model in predicting pathologic invasiveness of pulmonary nonsolid nodules. Radiology, 2025, 317(3): e251524. doi: 10.1148/radiol.251524 41432561

[43] Shao M, Wang J, Zhu L, et al. Deep learning for three-class classification of ground-glass nodules on non-enhanced chest CT: A multicenter comparative study of CNN architectures. Eur J Radiol Open, 2025, 15: 100690. doi: 10.1016/j.ejro.2025.100690 41127040 PMC12539229

[44] Pan Z, Hu G, Zhu Z, et al. Predicting invasiveness of lung adenocarcinoma at chest CT with deep learning ternary classification models. Radiology, 2024, 311(1): e232057. doi: 10.1148/radiol.232057 38591974

[45] Xu N, He Q, Wang L, et al. Predicting invasiveness of lung adenocarcinoma from chest CT with few-shot vision-language ternary classification model. NPJ Digit Med, 2025, 9(1): 60. doi: 10.1038/s41746-025-02229-2 41422131 PMC12820389

[46] Yu F, Peng M, Bai J, et al. Comprehensive characterization of genomic and radiologic features reveals distinct driver patterns of RTK/RAS pathway in ground-glass opacity pulmonary nodules. Int J Cancer, 2022, 151(11): 2020-2030. doi: 10.1002/ijc.34238 36029220 PMC9805018

[47] Kobayashi Y, Mitsudomi T, Sakao Y, et al. Genetic features of pulmonary adenocarcinoma presenting with ground-glass nodules: The differences between nodules with and without growth. Ann Oncol, 2015, 26(1): 156-161. doi: 10.1093/annonc/mdu505 25361983

[48] Nakamura R, Inage Y, Tobita R, et al. Epidermal growth factor receptor mutations: Effect on volume doubling time of non-small cell lung cancer patients. J Thorac Oncol, 2014, 9(9): 1340-1344. doi: 10.1097/jto.0000000000000022 24481317

[49] Yang Y, Yang Y, Zhou X, et al. *EGFR* L858R mutation is associated with lung adenocarcinoma patients with dominant ground-glass opacity. Lung Cancer, 2015, 87(3): 272-277. doi: 10.1016/j.lungcan.2014.12.016 25582278

[50] Zhou D, Li YQ, Liu QX, et al. Integrated whole-exome and bulk transcriptome sequencing delineates the dynamic evolution from preneoplasia to invasive lung adenocarcinoma featured with ground-glass nodules. Cancer Med, 2024, 13(11): e7383. doi: 10.1002/cam4.7383 38864483 PMC11167609

[51] Zhang JT, Zhang J, Wang SR, et al. Spatial downregulation of CD 74 signatures may drive invasive component development in part-solid lung adenocarcinoma. iScience, 2023, 26(10): 107699. doi: 10.1016/j.isci.2023.107699 PMC10550719 37810252

[52] Ren YF, Ma Q, Zeng X, et al. Single-cell RNA sequencing reveals immune microenvironment niche transitions during the invasive and metastatic processes of ground-glass nodules and part-solid nodules in lung adenocarcinoma. Mol Cancer, 2024, 23(1): 263. doi: 10.1186/s12943-024-02177-7 39580469 PMC11585206

[53] Xiong Y, Wang X, Yan K, et al. Epithelial plasticity shapes intratumoral heterogeneity and cell lineages in early-stage lung cancer. Sci Adv, 2026, 12(10): eady8546. doi: 10.1126/sciadv.ady8546 41790896 PMC12965311

[54] Tian Y, Ma R, Zhao W, et al. Comprehensive characterization of early-onset lung cancer, in Chinese young adults. Nat Commun, 2025, 16(1): 1976. doi: 10.1038/s41467-025-57309-4 PMC11861273 40000630

[55] Ortmeyer Welch K, McGovern KA, Chen L, et al. SGLT 2 inhibitor use reduces progression and surgical intervention of persistent pulmonary nodules. Lung Cancer, 2026, 215: 109356. doi: 10.1016/j.lungcan.2026.109356 41797126 PMC13470641

